# Nilotinib treatment outcomes in autosomal dominant spinocerebellar ataxia over one year

**DOI:** 10.1038/s41598-024-67072-z

**Published:** 2024-07-15

**Authors:** Woo-Jin Lee, Jangsup Moon, Yoonhyuk Jang, Yong-Woo Shin, Hyoshin Son, Seoyi Shin, Daejong Jeon, Dohyun Han, Soon-Tae Lee, Kyung-Il Park, Keun-Hwa Jung, Sang Kun Lee, Kon Chu

**Affiliations:** 1 Department of Neurology, Seoul National University College of Medicine, Seoul National University Hospital, 101, Daehangno, Jongno-gu, Seoul, 03080 Republic of Korea; 2 Department of Neurology, Seoul National University Bundang Hospital, Seongnam-si, Gyeonggi-do Republic of Korea; 3 Department of Neurology, Eunpyeong St. Mary’s Hospital, The Catholic University of Korea, Seoul, Republic of Korea; 4 Advanced Neural Technologies, Seoul, Republic of Korea; 5 Proteomics Core Facility, Biomedical Research Institute, Seoul National University Hospital, Seoul, Korea; 6 Department of Neurology, Seoul National University Hospital Healthcare System Gangnam Center, Seoul, South Korea; 7 Department of Neurology, Inha University Hospital, Incheon, Republic of Korea

**Keywords:** Neurology, Spinocerebellar ataxia

## Abstract

We evaluated the efficacy and safety of 1-year treatment with nilotinib (Tasigna^®^) in patients with autosomal dominant spinocerebellar ataxia (ADSCA) and the factors associated with responsiveness. From an institutional cohort, patients with ADSCA who completed a 1-year treatment with nilotinib (150–300 mg/day) were included. Ataxia severity was assessed using the Scale for the Rating and Assessment of Ataxia (SARA), scores at baseline and 1, 3, 6, and 12 months. A subject was categorized ‘responsive’ when the SARA score reduction at 12 M was > 0. Pretreatment serum proteomic analysis included subjects with the highest (n = 5) and lowest (n = 5) SARA score change at 12 months and five non-ataxia controls. Thirty-two subjects (18 [56.2%] females, median age 42 [30–49.5] years) were included. Although SARA score at 12 M did not significantly improve in overall population, 20 (62.5%) subjects were categorized as responsive. Serum proteomic analysis identified 4 differentially expressed proteins, leucine-rich alpha-2-glycoprotein (LRG1), vitamin-D binding protein (DBP), and C4b-binding protein (C4BP) beta and alpha chain, which are involved in the autophagy process. This preliminary data suggests that nilotinib might improve ataxia severity in some patients with ADSCA. Serum protein markers might be a clue to predict the response to nilotinib.

**Trial Registration Information:** Effect of Nilotinib in Cerebellar Ataxia Patients (NCT03932669, date of submission 01/05/2019).

## Introduction

Spinocerebellar ataxia (SCA) is a genetic disorder that leads to the progressive deterioration of cerebellar function^1–4^. Autosomal dominant spinocerebellar ataxia (ADSCA) is a major subgroup of SCA caused by the expansion of the trinucleotide repeat length in the gene and the abnormal elongation of the polyglutamine tract in the translated protein^1,2^. The common pathologic mechanism of ADSCA includes the aggregation of abnormally elongated proteins in neurons, which causes aberrant transcriptional regulation, mitochondrial dysfunction, synaptic neurotransmission disruption and cerebellar Purkinje cell degeneration^1–3^. Despite ongoing attempts to modulate the production of abnormal proteins, currently available treatment options for ADSCA remain at the level of symptom alleviation^1,5^.

Nilotinib (Tasigna® AMN107; Novartis, Switzerland) is a broad-spectrum tyrosine kinase inhibitor that preferentially targets the Abelson (Abl) and Discoidin domain receptors (DDRs)^6–8^. In Alzheimer’s disease (AD) and Parkinson’s disease (PD) models, both kinases are involved in the accumulation of pathogenic proteins in neurons, such as amyloid beta or alpha-synuclein^9,10^. In particular, Abl inhibits Parkin/Beclin-1-mediated promotion of autophagy while enhancing the PI3k/Akt/mTOR-mediated inhibition of autophagy^11–14^. In this regard, the effect of nilotinib has been reported in the preclinical models of Alzheimer’s disease and Parkinson’s disease by enhancing the autophagic clearance of protein aggregates^9–16^. Recently, two randomized controlled studies demonstrated the effect of nilotinib in patients with PD and in patients with AD^17,18^, although another randomized controlled study in in patients with moderately advanced PD reported a negative result^19^. Given that the main pathomechanism of ADSCA involves the chronic intracellular accumulation of abnormally elongated proteins, nilotinib might also be effective in improving ataxia symptoms and modifying disease progression in ADSCA^1–3^.

In our previous case series, nilotinib treatment was associated with clinical improvement in 12 patients with chronic cerebellar ataxia^20^. However, the retrospective design, small number of subjects, heterogeneous etiology of ataxia, lack of objective measurement for ataxia severity, and short duration of follow-up were the major limitations preventing the clinical application of the study results. In this study, we aimed to overcome those limitations by constructing an institutional prospective cohort of patients with cerebellar ataxia. Based on that, we evaluated the efficacy and safety of 1-year treatment with nilotinib in patients with ADSCA and demonstrated factors associated with its responsiveness.

## Methods

### Study population

From an ongoing institutional cohort of patients with cerebellar ataxia, 108 subjects with cerebellar ataxia who visited the Neurology Department of Seoul National University Hospital between May 2019 and March 2020 were initially included. Among them, the final study population included patients meeting the following criteria: (1) patients without a chronic heart, liver, kidney, or hematologic disorder that restricts the use of nilotinib; (2) patients who started regular administration of nilotinib with available baseline evaluations for ataxia severity; (3) patients whose ADSCA was confirmed as one of SCA1, SCA2, SCA3, SCA6, SCA7, SCA8, and SCA17^1,2^; and (4) patients who completed 1-year regular follow-up evaluations for ataxia severity^17,18,21^. The decision to start nilotinib treatment was made by the subjects and their caregivers after they were fully informed about the adverse effects and cost of nilotinib by the treating physician (K.C.). Before treatment initiation, checks for chronic cardiac, hepatic, or renal disorders were performed by evaluations of the subject’s medical history and laboratory parameters, including the complete blood count, liver enzyme panel, blood urea nitrogen and creatinine, serum electrolyte panel, and antibodies for chronic hepatitis viruses, and electrocardiography^8,17,18,20^. The dosage of nilotinib ranged from 150 to 300 mg per day, and the dosage was adjusted by the treating physician (K.C.) if clinically indicated, according to the clinical responsiveness and the subject’s tolerance to the medication^17,18,20^.

### Genetic testing for ADSCA

Genetic testing for ADSCA was performed using subjects’ serum with PCR analysis for the abnormal expansion in CAG repeats in the genes *ATXN1*, *ATXN2*, *ATXN3*, *CACNA1A*, and ATXN7; CAG/CTG repeats in *ATXN8*; and CAG/CAA repeats in *TBP*. For each gene, the cutoff for the trinucleotide repeat number for the full penetrance of disease was set as 39 for *ATXN1*, 37 for *ATXN2*, 60 for *ATXN3*, 20 for *CACNA1A*, 37 for *ATXN7*, 54 for *ATXN8*, and 49 for *TBP*^1,2^.

### Acquisition of clinical and laboratory data

Along with demographic information, clinical and laboratory information, including the age at the onset of the clinical symptoms of cerebellar ataxia, maintenance dosage of nilotinib, and the length of trinucleotide repeats in the allele of longer repeat length, were obtained. The severity of ataxia and the ability to perform activities of daily living (ADLs) were assessed at baseline and at 1, 3, 6, and 12 months (− 1 M, − 3 M, − 6 M, and − 12 M, respectively) after the initiation of nilotinib administration. At every follow-up visit, the subjects were monitored for adverse drug reactions to nilotinib and underwent evaluations for a complete blood count, liver enzyme panel, blood urea nitrogen and creatinine, and serum electrolyte panel as well as electrocardiography^8,17,18,20^.

The clinical severity of ataxia was assessed using the Scale for the Rating and Assessment of Ataxia (SARA) scores (8 items; score range, 0–100, where a higher score indicates a higher severity of ataxia)^17,18,22^. The SARA score was measured by two neurology experts (W.-J.L. and Y.-H.J. or Y.-W.S.). For the discrepant cases, consensuses were reached after a review of the videotape and discussion. Inter-rater (W.-J.L. and Y.-H.J. or Y.-W.S) agreement for SARA score assessed through separate reviews of the video recordings for the SARA score evaluation at each time points was high (0.82). The changes in ADL functioning were assessed using a battery of structured questionnaires, which included the functional stage subscale of the Friedreich Ataxia Rating Scale (FARS I; score range, 0–6, with a higher score indicating a more advanced stage of disease), the ADL subscale of the FARS (FARS II; score range, 0–36, with a higher score indicating more impaired ADL functioning), and the modified version of the Barthel Index (BI; score range, 0–100; with a higher score indicating a higher degree of independence)^23,24^.

### Outcome analysis

The main outcome was the change in the SARA score from baseline. Subjects were categorized into the ‘responsive’ group when the SARA score change-12 M (SARA score at baseline minus the SARA score at 12 M) was > 0 or into the ‘nonresponsive’ group when the SARA score change-12 M was ≤ 0. The changes in the FARS I, FARS II, and BI scores were also measured. Adverse events were classified according to the Common Terminology Criteria for Adverse Events (CTCAE v5.0)^25^. A severe adverse event was designated as an adverse event of CTCAE grade 3 or higher.

### Serum proteomic analysis

Five subjects with the highest SARA score change-12 M scores in the responsive (R) group and five subjects with the lowest SARA score change-12 M scores in the nonresponsive (N) group were selected for the serum proteomic analysis. Serum samples were obtained at the time of laboratory check-up before the initiation of nilotinib treatment. As a control (C) group, five subjects with recurrent dizziness and a similar age range to those of the patient groups but without evidence of ataxia or chronic central nervous system disease were selected from our serum repository database^26,27^.

Serum proteomic analysis was performed using 200 µl of serum samples stored at − 80 °C. The detailed process of proteomic analysis was described in previous reports^28–30^. In brief, the process included serum sample preparation, liquid chromatography–tandem mass spectrometry (LC–MS/MS) analysis using a Q-exactive HF-X (Thermo Fisher Scientific, Waltham, MA, USA) coupled to an Ultimate 3000 RSLC system (Dionex, Sunnyvale, CA, USA), and data processing for label‑free quantification. Mass spectra were processed in MaxQuant software (ver. 1.6, Max-Planck Institute, Munich, Germany), and MS/MS spectra were searched against the Human UniProt protein sequence database. The required false discovery rate (FDR) for all proteins and peptides was set as 1%.

The analysis of the differentially expressed proteins (DEPs) was performed using Perseus software^30^. The expression level of proteins in the serum was estimated by the intensity-based absolute quantification values using MaxQuant software. After log2 transformation of those values, proteins with less than three quantified values in each group were filtered out, and missing values were imputed based on a normal distribution (width = 0.3 and downshift = 1.8). Analysis of variance (ANOVA) was performed to detect DEPs among the groups with a significance level of *P* < 0.05. DEPs were z-normalized for their abundances followed by hierarchical clustering with Euclidean distance. For the adjustment for multiple comparisons, the Benjamini‒Hochberg method was used with the Benjamini‒Hochberg critical value calculated using the false discovery rate of 0.2^31^.

### Statistical analysis

R software version 4.0.3 (2021; R team, Vienna, Austria) was used for all statistical analyses. Data are reported as numbers (percentage), mean ± standard deviation, or median (range). For the comparison between responsive and nonresponsive groups, a two-tailed Student’s t test, Pearson’s chi-square, Kruskal‒Wallis, or Mann‒Whitney test were used. Spearman’s rho was used for the correlation analyses between the SARA score and the FARS I, FARS II, and BI scores. To evaluate the change in the SARA, FARS I, FARS II, and BI scores from baseline, repeated-measure analysis of covariance (RM-ANCOVA) adjusting for age, sex, type of SCA, baseline score, duration of disease, maintenance dosage of nilotinib, and length of the trinucleotide repeat was performed. For every analysis, a *P* value of < 0.05 was considered statistically significant.

### Standard protocol approvals, registrations, and patient consents

The study procedures were approved by the institutional review board of the Seoul National University Hospital. All methods were performed in accordance with the STROBE guidelines for cohort studies, and the 2013 amended version of the Declaration of Helsinki. The protocols of the study were registered in clinicaltrials.gov (Effect of Nilotinib in Cerebellar Ataxia Patients, registration number: NCT03932669, date of registration: 01/05/2019). Written informed consent was obtained from all study participants. The study protocol and statistical analysis plan are available in Supplemental Dataset.

## Results

### Patient characteristics

**Figure 1:**
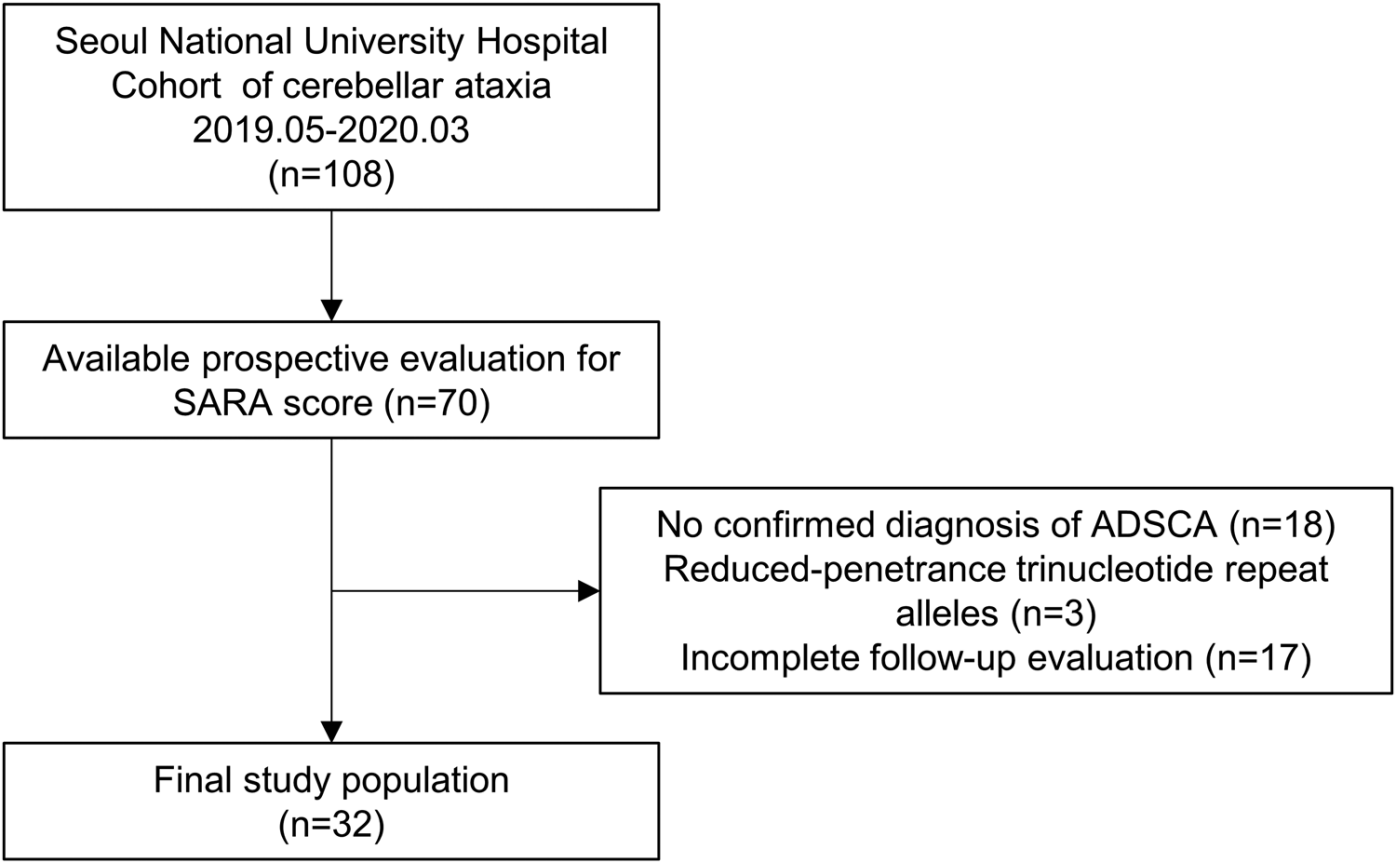
Study population SARA: Scale for the Assessment and Rating of Ataxia and ADSCA: autosomal dominant spinocerebellar ataxia.

Among the initially included 108 subjects with cerebellar ataxia, 70 subjects without a chronic heart, liver, kidney, or hematologic disorder and with available baseline evaluations for ataxia severity and started regular administration of nilotinib. Among them, 18 subjects without a confirmed diagnosis of ADSCA, three subjects with a trinucleotide repeat number of reduced-penetrance trinucleotide alleles, and 17 patients who did not complete 1-year maintenance therapy with nilotinib and regular follow-up evaluations were sequentially excluded, and the remaining 32 subjects (14 [43.8%] males and 18 [56.2%] females, median age 42 [30.5–49.5] years) were included in the final analysis (Fig. 1). Eight (25.0%) subjects had SCA2, 10 (31.2%) had SCA3, 8 (25.0%) had SCA6, 3 (9.4%) had SCA7, 2 (6.2%) had SCA 8, and 1 (3.1%) had SCA 17. The median age of onset was 44 [31–50] years, and the median duration of disease from symptom onset was 4.5 [2–8] years. At baseline, the median SARA score was 10.5 [8.8–13.2], the median FARS I score was 2 [2], the median FARS II score was 9.5 [6.5–13], and the median BI was 1.5 [0–3].

### Efficacy of nilotinib in *ataxia* symptoms

The maintenance dosage of nilotinib was 150 mg/day in six (18.8%), 200 mg/day in three (9.4%), and 300 mg/day in 23 (71.9%) subjects. The median SARA score change-12 M was 1.5 [− 0.5–2.2], and 20 (62.5%) subjects were categorized as responsive. The demographic characteristics, frequencies of SCA types, trinucleotide repeat length, onset age, baseline scores for ataxia severity, and maintenance dosage of nilotinib were similar between the responsive and nonresponsive groups (Table 1). Among the 17 subjects who did not complete follow-up, the reasons for discontinuation were a lack of subjective improvement in ataxia symptoms in four subjects, adverse events in three subjects (two with dizziness and one with stomach pain, all CTCAE grade 1), and the cost of the drug in three subjects, while in the remaining seven subjects, the reason was not identified.

**Table 1:** Comparison between the subjects with or without clinical improvement Clinical, and cerebrospinal fluid profiles of the patients.

	Total (N = 32)	Responsive (N = 20)	Non-reponsive (N = 12)	*P*
Sex (men, %)	14 (43.8%)	10 (50.0%)	4 (33.3%)	0.581
Age (years)	42 [30.5–49.5]	39.5 [30.5–46]	48.5 [30.5–59]	0.267
Type				0.389
SCA2 (%)	8 (25.0%)	5 (25.0%)	3 (25.0%)	
SCA3 (%)	10 (31.2%)	4 (20.0%)	6 (50.0%)	
SCA6 (%)	8 (25.0%)	6 (30.0%)	2 (16.7%)	
SCA7 (%)	3 (9.4%)	3 (15.0%)	0 (0.0%)	
SCA17 (%)	2 (6.2%)	1 (5.0%)	1 (8.3%)	
SCA8 (%)	1 (3.1%)	1 (5.0%)	0 (0.0%)	
Trinucleotide repeat length (long allele)	45 [39–70.5]	45 [32–67.5]	66 [40.5–70.5]	0.447
SCA2 (n = 8)	43 [41.25–45]	45 [41. 5–45.5]	42 [39–44]	0.292
SCA3 (n = 10)	71.5 [67.0–73.25]	72.5 [72.0–73.75]	68.5 [66.5–72.25]	0.089
SCA6 (n = 8)	23.5 [22.25–25.0]	24 [22.75–28.5]	23.0	0.301
SCA7 (n = 3)	45 [42–53]	45 [42–53]	–	–
SCA8 (n = 2)	94.5	81	108	–
SCA17 (n = 1)	63	63	–	–
Years from symptom onset (years)	4.5 [2–8]	3 [2–5.5]	5.5 [2.5–9]	0.169
Dosage of nilotinib				0.329
150 mg/day (%)	6 (18.8%)	5 (25.0%)	1 (8.3%)	
200 mg/day (%)	3 (9.4%)	1 (5.0%)	2 (16.7%)	
300 mg/day (%)	23 (71.9%)	14 (70.0%)	9 (75.0%)	
Baseline severity				
SARA score	10.5 [8.8–13.2]	10.8 [9.5–14.5]	9.8 [8–11.8]	0.283
FARS I score	2 [2–2]	2 [2–2]	2 [2–2.5]	0.311
FARSII score	9.5 [6.5–13]	8.5 [5.5–13.5]	10.5 [9–12]	0.482
Barthel index	1.5 [0–3]	1 [0–4]	2 [0–3]	0.734
Follow-up evaluations				
SARA score change-1 M	0.5 [− 1–0.8]	0.5 [− 0.5–2]	-0.5 [− 1–− 0.2]	0.017*
SARA score change-3 M	1 [0–1.5]	1.5 [1–3]	− 0.5 [− 1–0.5]	< 0.001**
SARA score change-6 M	1 [− 0.8–2]	2 [1.2–2.8]	− 0.8 [− 1.5–− 0.5]	< 0.001**
SARA score change-12 M	1.5 [− 0.5–2.2]	2 [1.5–3]	− 0.8 [− 2–− 0.2]	< 0.001**
FARS I score change-1 M	0 [0–0]	0 [0–0]	0 [0–0]	1.000
FARS I score change-3 M	0 [0–0]	0 [0–1]	0 [0–0]	0.737
FARS I score change-6 M	0 [0–1	0 [0–1]	0 [0–1]	0.851
FARS I score change-12 M	0 [0–0]	0 [0–0]	0 [0–1]	0.442
FARS I score improvement (%)	8 (25.0%)	5 (25.0%)	3 (25.0%)	1.000
FARS II score change-1 M	1 [0–2]	1 [0–2]	0.5 [− 0.5–2]	0.553
FARS II score change-3 M	1 [0–2.5]	1.5 [0–3]	0.5 [− 0.5–2]	0.237
FARS II score change-6 M	1 [0–3]	1 [0–3.5]	0 [− 0.5–2]	0.333
FARS II score change-12 M	1 [0–3]	1 [− 1–2]	1.5 [0–3.5]	0.399
FARS II score improvement (%)	20 (62.5%)	12 (60.0%)	8 (66.7%)	1.000
Barthel index change-1 M	0 [0–1]	0 [0–1]	0 [0–0]	0.207
Barthel index change-3 M	0 [0–1]	0 [0–1]	0 [− 0.5–0]	0.017*
Barthel index change-6 M	0 [0–0]	0 [0–0.5]	0 [− 1–0]	0.098
Barthel index change-12 M	0 [0–0.5]	0 [0–1]	0 [− 0.5–0]	0.411
Barthel index improvement (%)	8 (25.0%)	6 (30.0%)	2 (16.7%)	0.673

Data are reported as a number (percentage) or median [interquartile range, IQR].

*SCA* spinocerebellar ataxia, *SARA* scale for the assessment and rating of ataxia, *FARS* Rating scale for Friedreich’s ataxia.

*P < 0.05 and **P < 0.01.

Although the SARA score at baseline exhibited a moderate correlation with the baseline scores of the FARS I, FARS II, and BI (all, *P* < 0.001), the correlation of the change in the SARA score with the changes in those scores was weak (Supplemental Table 1 and Supplemental Fig. 1). Adverse events were observed in 15 (42.9%) subjects, nine with transient and mild alanine aminotransferase increases, two with gastrointestinal discomfort, one with mild folliculitis, one with urticaria, one with dizziness, and one with headache (all CTCAE grade 1).

**Figure 2:**
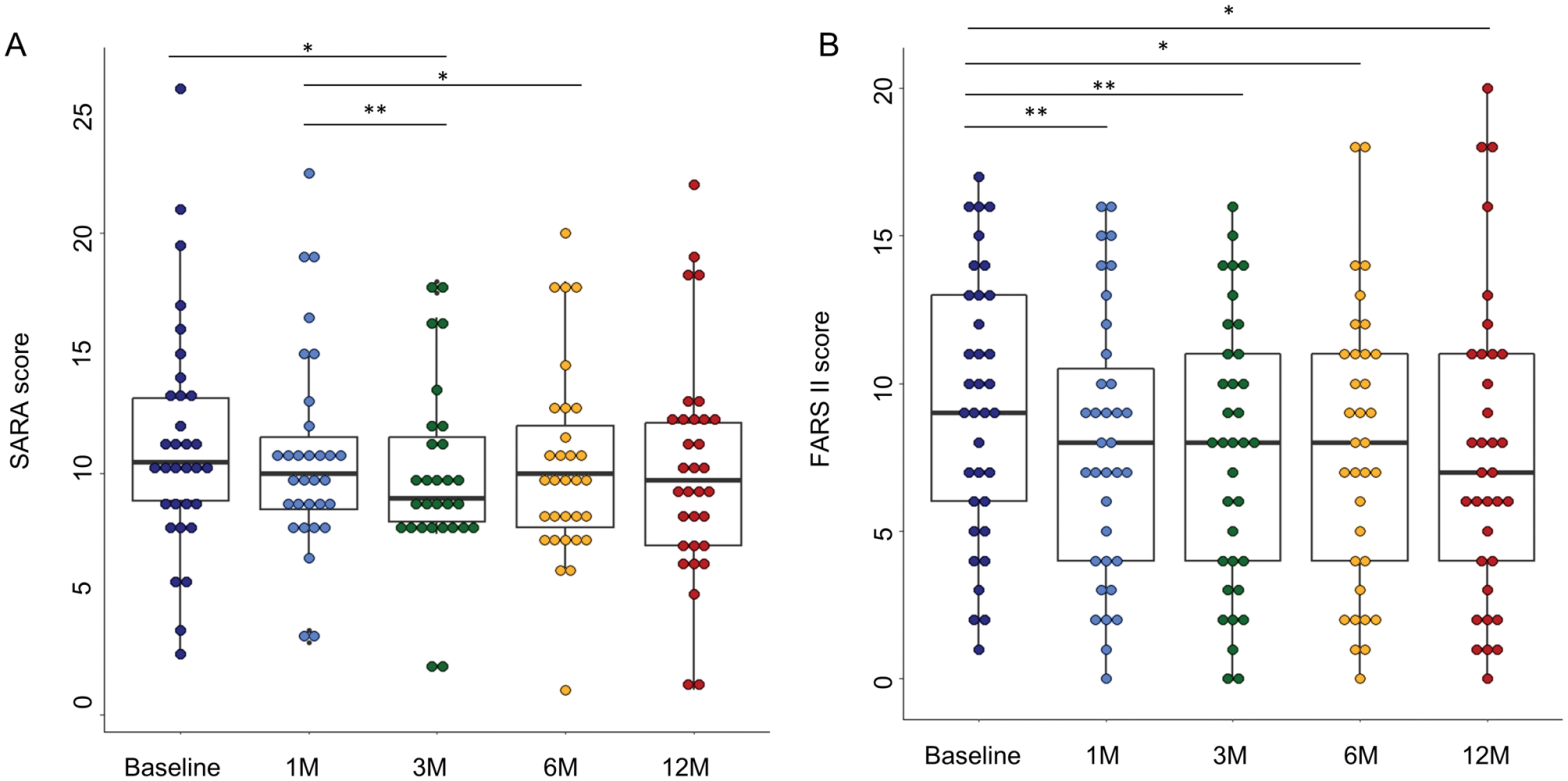
Box and whisker plots for the 1-year changes in the measurements of the clinical severity of ataxia 1-year changes in the SARA (**A**) and FARS II (**B**). SARA: Scale for the Assessment and Rating of Ataxia, and FARS: Friedreich Ataxia Rating Scale. The upper and lower margins of the rectangle represent the first and third quartiles, respectively. The horizontal line within the box represents the median value. The upper and lower vertical lines represent ranges or distances of 1.5 times the interquartile range from the first or third quartile values, respectively. ^*^P < 0.05.

**Figure 3:**
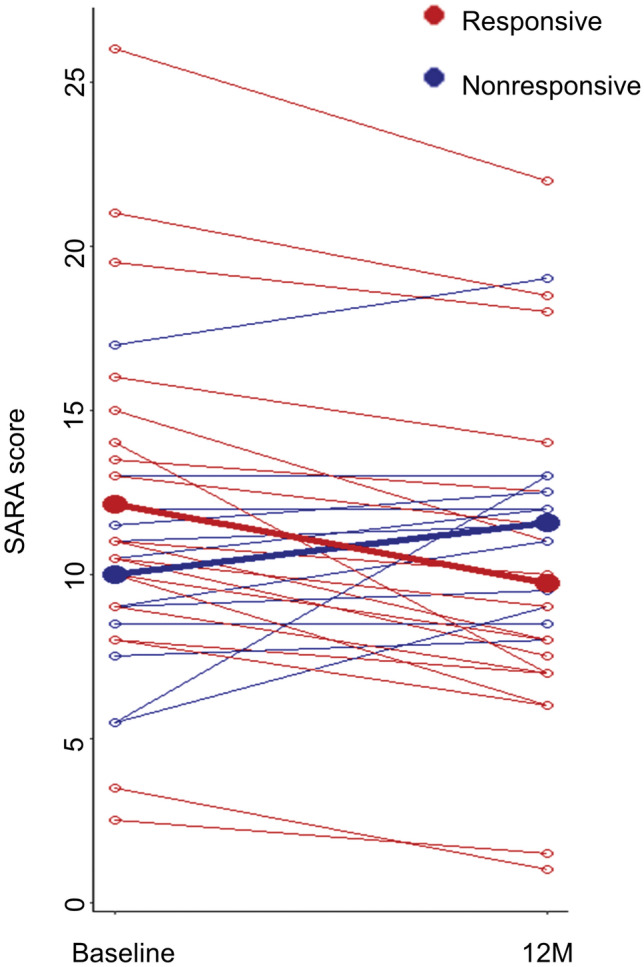
Spaghetti plot for SARA score changes at 12-month from the baseline. Patients in the responsive group were marked in red, whereas patients in the nonresponsive group were marked in blue. Thick lines with solid points indicate the mean SARA scores and the changes in each group.

SARA score at 3 M exhibited a significant improvement compared to the baseline and to 1 M (*P* = 0.011 and *P* = 0.001, respectively) and SARA score at 6 M showed a higher improvement compared to the 1 M (*P* = 0.041). However, SARA score at 12 M did not exhibit a significant improvement compared to the baseline (*P* = 0.058, Fig. 2A). In RM-ANCOVA, the SARA score showed significant improvement from baseline (F = 2.595, *P* = 0.039), and higher SARA scores at baseline (F = 589.207, *P* < 0.001) and SCA subtypes (F = 2.636, *P* = 0.026, more improved SARA scores in SCA7 than in SCA6) were associated with better improvement in the SARA score (Table 2). In the spaghetti plot for 12 M SARA score changes, no significant difference in the baseline SARA score was observed between the responsive and nonresponsive groups (Fig. 3). The FARS II scores also significantly improved over time (F = 2.612, *P* = 0.038), and the FARS II scores at each evaluation time point (1 M, 3 M, 6 M, or 12 M) showed a significant improvement compared to baseline (*P* = 0.008, *P* = 0.006, *P* = 0.039, and *P* = 0.025, respectively) (Table 2 and Fig. 2B). Neither the FARS I score, nor the BI score exhibited a significant improvement compared to baseline (Supplemental Table 2Supplemental Fig. 2).

**Table 2:** Repeated measure ANCOVA analysis for the SARA and FARS II score changes.

	DF	Sum Square	Mean Square	F value	*P*	Post-Hoc
SARA score change						
Residuals		519.9	3.7			–
Follow-up (months)	4	38.8	9.7	2.595	0.039*	3 M > baseline*, 3 M > 1 M**, 6 M > 1 M*
SARA score at baseline	1	2204.0	2204.0	589.207	< 0.001**	–
Age (years)	1	17.2	17.2	4.601	0.034*	–
Male sex	1	7.8	7.8	2.077	0.152	
Type	5	49.3	9.9	2.636	0.026*	SCA7 > SCA6**
Trinucleotide repeat length (long allele)	1	3.8	3.8	1.009	0.317	–
Duration of disease (years)	1	10.4	10.4	2.786	0.097	–
Dosage of nilotinib (mg)	1	11.5	11.5	3.074	0.082	–
FARS II score change						
Residuals		1029.1	7.2			–
Follow-up (months)	4	75.7	18.9	2.612	0.038*	1 M, 3 M > Baseline** 6 M, 12 M > Baseline*
SARA score at baseline	1	1754.0	1754.0	242.036	< 0.001**	–
Age (years)	1	58.0	58.0	8.008	0.005**	–
Male sex	1	0.2	0.2	0.028	0.867	
Type	5	89.9	18.0	2.481	0.035*	SCA6 > SCA2**, SCA6 > SCA3*
Trinucleotide repeat length (long allele)	1	0.3	0.3	0.044	0.835	–
Duration of disease (years)	1	1.6	1.6	0.214	0.644	–
Dosage of nilotinib (mg)	1	1.4	1.4	0.196	0.658	–

*ANCOVA* analysis of covariance, *DF* degree of freedom, *SARA* scale for the assessment and rating of ataxia, *FARS* Rating scale for Friedreich’s ataxia, *SCA* spinocerebellar ataxia.

*P < 0.05 and **P < 0.01.

### DEPs associated with the responsiveness to nilotinib

**Figure 4:**
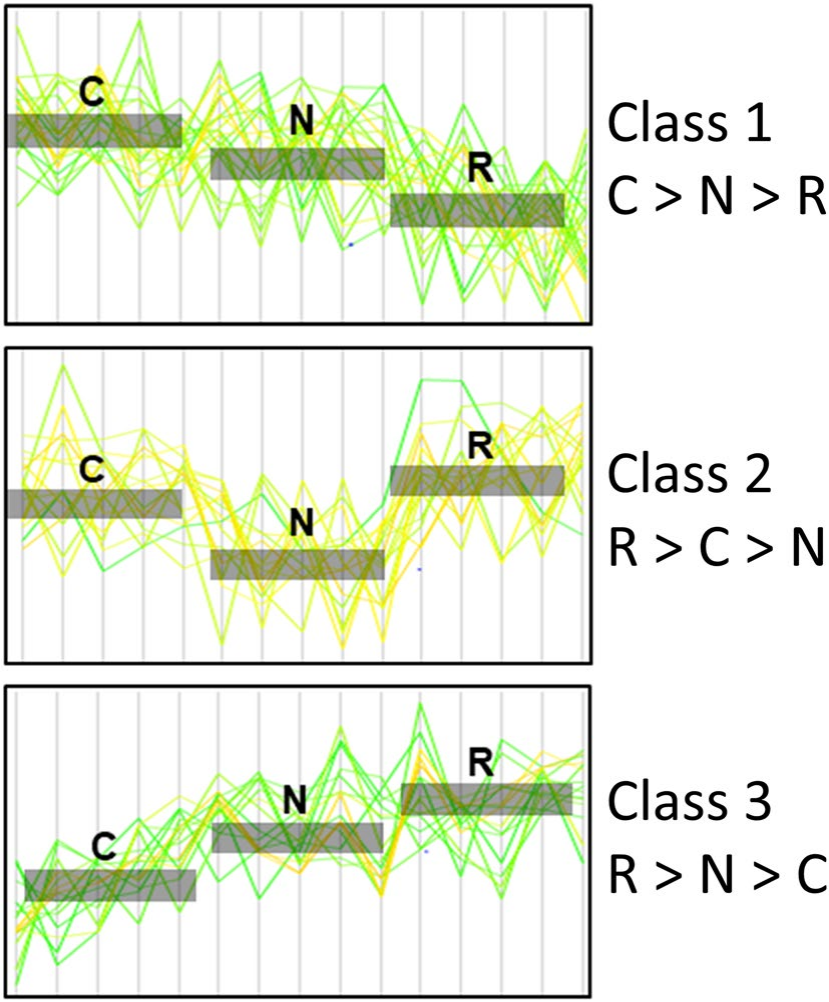
Classification of the DEPs according to their expression patterns. Differentially expressed proteins (DEPs) were classified into 5 classes according to their expression pattern. Class 1 exhibits the lowest expression level in the subjects responsive to nilotinib (subgroup R), an intermediate level in the subjects nonresponsive to nilotinib (subgroup N), and the highest expression level in the control subjects (subgroup C). Class 2 exhibits the lowest expression level in subgroup N, an intermediate level in subgroup C, and the highest expression level in subgroup R. Class 3 exhibits the lowest expression level in subgroup C, an intermediate level in subgroup N, and the highest expression level in subgroup R.

Serum proteomic analysis covering 895 proteins identified 50 DEPs, which were further classified into 3 classes as follows: class 1 (C > N > R, 22 DEPs), class 2 (R > C > N, 13 DEPs), and class 3 (R > N > C, 15 DEPs) (Fig. 4, list of DEPs in Supplemental Table 3, and full proteomic analysis data in Supplemental Dataset). The profiles of subjects and control subjects included in the proteomic analysis are summarized in Supplemental Table 4.

After Benjamini‒Hochberg adjustment for multiple comparisons, 4/50 (8.0%) proteins remained differentially expressed among the groups, including leucine-rich alpha-2-glycoprotein (LRG1) in class 1, vitamin-D binding protein (DBP) in class 2, and C4b-binding protein (C4BP) beta and alpha chain in class 3 (Table 3). A literature search revealed that all 4 proteins are involved in the autophagy process. Remarkably, DEPs with the lowest expression in the R group had a promotive effect on autophagy, whereas DEPs with the highest expression in the R group had an inhibitory effect on autophagy. LRG1 promotes autophagy via the regulation of the TGF-β-smad1/5 signaling pathway^32^; DBP inhibits autophagy by sequestrating free vitamin D, which enhances autophagy via mTOR inhibition at the induction stage, Beclin1 and PI3K3 augmentation at the nucleation stage, and the activation of lysosomes at the maturation and degradation stage of autophagy^33–35^; and C4BPs inhibit C3 and C4 binding, which activates the autophagy machinery^36,37^; and inhibit protein S (PS) by binding to PS, blocking the site of PS responsible for the effect on the phagocytic index^38^.

**Table 3:** Differentially expressed proteins among the serum of the subgroups.

Classes	Protein description	Name	Unadjusted *P*	Benjamini–Hochberg critical value	Effect on autophagy	Functions
Class 1 C > N > R	Leucine-rich alpha-2-glycoprotein	LRG1	0.0001	0.0002*	Enhancement	Promote autophagy via the regulation of the TGF-β-smad1/5 signaling pathway
Class 2 R > C > N	Vitamin-D binding protein	VDB	0.0002	0.0002*	Inhibition	Sequestrates free vitamin D, which enhances autophagy via mTOR inhibition at the induction stage, Beclin 1 and PI3K3 augmentation at the nucleation stage, and activation of lysosome at the maturation and degradation stage
Class 3 R > N > C	C4b-binding protein beta chain	C4BPB	0.0002	0.0002*	Inhibition	C4b-binding protein (C4BP), composed of seven alpha-chains and one beta-chain, is main soluble regulator of complement system Binds with protein S (PS), blocking the site of PS responsible for the effect on phagocytic index
Class 3 R > N > C	C4b-binding protein alpha chain	C4BPA	0.0004	0.0004*		

*C* control, *N* non-responsive to nilotinib, *R* responsive to nilotinib.

Unadjusted *P* valued were derived from the analysis of covariance. Benjamini–Hochberg adjustment was used for the adjustment for the multiple comparison. Benjamini–Hochberg critical value was calculated using the false discovery rate of 0.2

*Statistical significance after Benjamini–Hochberg adjustment.

## Discussion

In this prospective cohort study, we observed an improvement in ataxia symptoms during treatment with nilotinib in patients with ADSCA. Although SARA score at 12 M did not significantly improve in overall population, an improvement in SARA scores at 1 year was observed in 62.5% of subjects, while nilotinib was not associated with severe adverse events in most subjects. Serum proteomic analysis revealed 4 DEPs that were associated with the responsiveness to nilotinib, all of which are involved in the regulation of autophagy process. Additionally, age, sex, trinucleotide repeat length, onset age, and maintenance dosage of nilotinib were not significantly associated with responsiveness, whereas the SCA7 subtype was associated with better improvement than the SCA6 subtype. Although a previous study reported a nilotinib-associated improvement in ataxia symptoms in a small number of subjects with chronic cerebellar ataxia of heterogeneous etiology based on a retrospective design^20^, this is the first prospective study to demonstrate that nilotinib might improve ataxia symptoms in ADSCA.

In this study, nilonitib was associated with a median 1.5-point decrement in the SARA score at one year. Although not statistically significant, the modest improvement in SARA score might be of clinical significance, considering that the annual increase in the SARA score reported in a large-sized cohort study ranged from 0.8 to 2.1 points according to the subtypes^21^. The efficacy and tolerability of nilotinib in patients with ADSCA in this study are in concordance with the previous randomized studies that evaluated the effect of nilotinib in PD and in AD^17,18^. In those studies, 1-year maintenance treatment with 150–300 mg/day of nilotinib resulted in a significant reduction in the cerebrospinal fluid (CSF) level of hyperphosphorylated tau and an incremental change in the CSF level of dopamine metabolites in PD as well as a reduction in the CSF level of phosphorylated-tau-181 and amyloid beta 40 and 42 and the rate of hippocampal volume loss in AD^17,18^. Considering that both PD and AD share common pathological characteristics with ADSCA^9,10^, the effect of nilotinib on ADSCA might also be explained by its autophagy-enhancing property of the abnormally accumulated intraneuronal protein caused by trinucleotide repeat expansions. However, the clinical evidence regarding the efficacy of nilotinib is mixed. A recent randomized study reported a lack of clinical efficacy, change in the dopamine metabolites in the CSF in patients with moderately advanced PD^19^. More advanced status of disease associated with progressed degeneration of dopaminergic neurons might have lowered the chance of improvement by nilotinib, which might be an explanation for the negative result. This is in concordance with that the current study did not observe a significant improvement in 12-month SARA score in the whole study population. Nevertheless, improvement of SARA scores was observed in 62.5% patients, indicating that optimal selection of patient who would have clinical improvement by nilotinib might be important.

In this regard, it is remarkable that proteomic analysis of the pretreatment serum identified 4 DEPs associated with the responsiveness to nilotinib. Especially, DEPs elevated the R group inhibit autophagy while the decreased DEPs enhance autophagy. This finding indicates that (1) autophagy system function might be dysregulated in ADSCA, with subject-to-subject variability in the pattern and degree of dysregulation; (2) nilotinib might improve ataxia symptoms mainly by enhancing or restoring autophagy system function; and (3) the serum proteome might serve as a biomarker that predicts clinical response to nilotinib. For instance, C4BP is a major regulator of the complement system. C4BP inhibits C4 and C3b and accelerates the decay of C3-convertase, while C3 and C4 binding activates the autophagy machinery^36,37^. C4BP also inhibits the phagocytosis of damaged cells by binding with the autophagy inducer PS and blocking PS from binding to the PS receptor on phagocytic cells^38^. DBP inhibits autophagy by sequestrating free vitamin D and vitamin D receptor (VDR), which enhances macroautophagy via mTOR inhibition, beclin1 and PI3K3 augmentation, and lysosome activation^33–35^. Thus, the elevation of C4BP and DBP in the R group might indicate an inhibited complement-mediated autophagy system and vitamin D- and VDR-mediated augmentation of autophagy in this group, leading to a higher chance of the enhancement of the autophagy function by nilotinib. In contrast, the low level of LRG1 in the R group, a protein that promotes autophagy by regulating the TGF-β-smad1/5 signaling pathway, might indicate a lower chance of the restoration of autophagy function by nilotinib^32^.

The SCA7 subtype was associated with a higher SARA score change than the SCA6 subtype. This should not be interpreted as SCA7 being more responsive to nilotinib, as the number of subjects with that subtype was small SCA7 (n = 3). Additionally, the correlation of ataxia severity changes measured using the SARA with the self-reported changes in ADL functioning was low. Non-ataxia symptoms of ADSCA, possible placebo or psychological effects, and possible adaptation to the task items of the SARA might be possible explanations for this low correlation^21,39–41^.

The current study has some limitations. First, as an observation study that included a small number of subjects, this study provides a low level of evidence for the efficacy of nilotinib in the treatment of ADSCA. Although we performed post-hoc analyses for the nilotinib effect in different subtypes of ADSCA, this adjustment is limited considering the heterogeneous pathomechanisms among the subtypes which are incorporated as a single study population. As a non-randomized study, factors such as unblinded clinical assessment, the practice effect associated with SARA scoring, and the unrandomized and non-standardized protocol for adjusting nilotinib doses might be limitations that warrants careful interpretation of study result. The frequency of improvement with nilotinib should be interpreted with caution, as the number of subjects who did not complete the 1-year follow-up was considerably high. Although not fully investigated, the major reason for dropping out might have been an insufficient clinical response to the drug, and the rate of clinical improvement might be considerably lower when the dropped-out patients were included in the intention-to-treat analysis. Although the number of trinucleotide repeats and their clinical implication are highly variable according to the ADSCA subtypes, we were unable to standardize the burden of trinucleotide repeat length among the subtypes. This study did not validate the association of the 4 DEPs with the clinical responsiveness in the whole study population, which might largely limit the efficacy of those serum biomarkers for predicting the responsiveness to nilotinib. Additionally, this study did not include the CSF biomarkers assessment of neuronal damage, such as tau protein or neurofilament light chain^17,18,42^. Further large-sized studies with long-term follow-up, including serum and CSF proteomic analyses to confirm the association of the DEPs in predicting the clinical responsiveness, are expected to be available for this cohort, and future randomized controlled studies are also warranted to address those issues.

## Data availability

The datasets generated during and/or analyzed during the current study are available from the corresponding author on request.

### Supplementary Information

Supplementary Information 1.

Supplementary Information 2.
