# Supplementary material for: Nilotinib treatment outcomes in autosomal dominant spinocerebellar ataxia over one year
Source: Sci Rep. 2024 Jul 15;14:16303. doi: 10.1038/s41598-024-67072-z (PMC11251258; doi:10.1038/s41598-024-67072-z)
Supplement: Supplementary file 1 — Supplementary Information 1. [file 41598_2024_67072_MOESM1_ESM.docx]

Supplemental Materials

**Nilotinib Treatment Outcomes in Autosomal Dominant Spinocerebellar Ataxia Over One Year**

**List of Supplements**: 2 Supplemental Figures and 4 Supplemental Tables

Supplemental Fig 1. Correlation of SARA score with other measurements for the clinical severity of ataxia

Supplemental Fig 2. Box and whisker plots for the 1-year changes in the measurements of the clinical severity of ataxia

Supplemental Table 1. Correlation among SARA, FARS I, FARS II, and Barthel index scores and their changes

Supplemental Table 2. Repeated measure ANCOVA analysis for the FARS I and Barthel index score changes

Supplemental Table 3. Differentially expressed proteins among the serum of the subgroups

Supplemental Table 4. Subjects with serum proteomic analysis

**Supplemental Fig 1. Correlation of SARA score with other measurements for the clinical severity of ataxia**


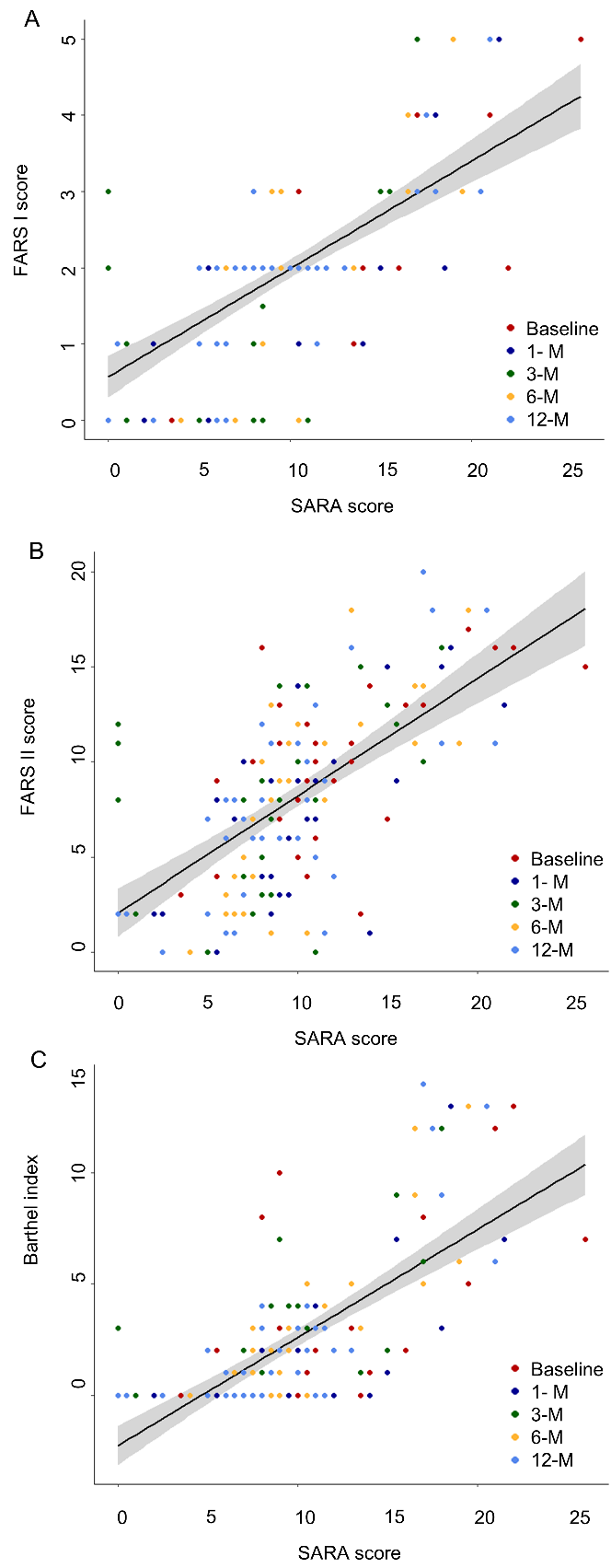


Correlation of SARA score with FARS I (**panel A**), FARS II (**panel B**), and Barthel index (**panel C**) scores. SARA: scale for the assessment and rating of ataxia, and FARS: Rating scale for Friedreich’s ataxia. ^*^*P*<0.05 and ^**^*P*<0.01.

**Supplemental Fig 2. Box and whisker plots for the 1-year changes in the measurements of the clinical severity of ataxia**


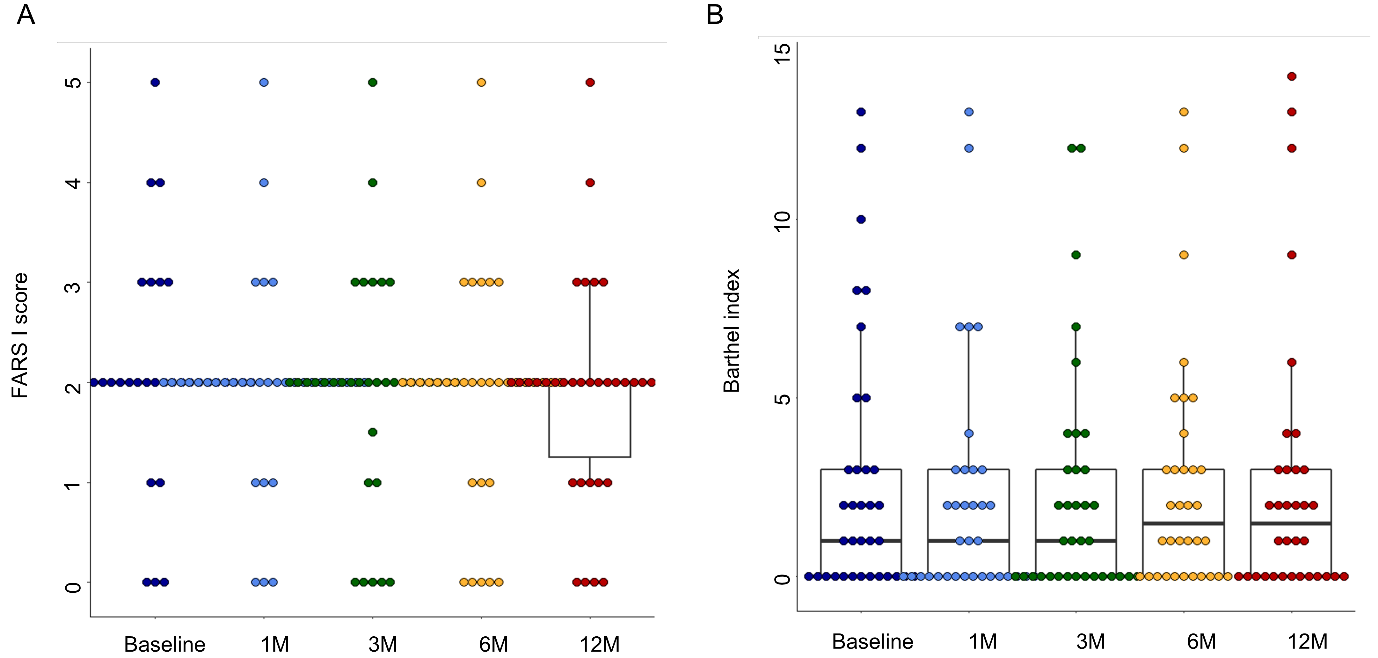


1-year changes in the FARS I (A) and Barthel index (B) scores. FARS: Friedreich Ataxia Rating Scale. The upper and lower margins of the rectangle represent the first and third quartiles, respectively. The horizontal line within the box represents the median value. The upper and lower vertical lines represent ranges or distances of 1.5 times the interquartile range from the first or third quartile values, respectively. *P<0.05.

**Supplemental Table 1. Correlation among SARA, FARS I, FARS II, and Barthel index scores and their changes**

|  | **Spearman's rho** | ***P*** |
| --- | --- | --- |
| **SARA score at baseline** |  |  |
| FARS I score at baseline | 0.569 | <0.001^**^ |
| FARS II score at baseline | 0.603 | <0.001^**^ |
| Barthel index at baseline | 0.589 | <0.001^**^ |
| **SARA score changes** |  |  |
| FARS I score changes | 0.250 | 0.004^**^ |
| FARS II score changes | 0.124 | 0.154 |
| Barthel index changes | 0.227 | 0.008^**^ |

SARA: scale for the assessment and rating of ataxia, and FARS: Rating scale for Friedreich’s ataxia. ^*^*P*<0.05 and ^**^*P*<0.01.

**Supplemental Table 2. Repeated measure ANCOVA analysis for the FARS I and Barthel index score changes**

|  | **DF** | **Sum Square** | **Mean Square** | **F value** | ***P*** | **Post-Hoc** |
| --- | --- | --- | --- | --- | --- | --- |
| FARS I score change |  |  |  |  |  |  |
| Residuals |  | 24.3 | 0.2 |  |  |  |
| Follow-up (months) | 4 | 1.1 | 0.3 | 1.662 | 0.162 |  |
| SARA score at baseline | 1 | 127.8 | 127.8 | 745.981 | <0.001^**^ |  |
| Age (years) | 1 | 1.1 | 1.1 | 6.509 | 0.012^*^ |  |
| Male sex | 1 | 0.4 | 0.4 | 2.216 | 0.139 |  |
| Type | 5 | 2.7 | 0.6 | 3.195 | 0.009^**^ | SCA6> SCA2^*^ |
| Trinucleotide repeat length (long allele) | 1 | 0.0 | 0.0 | 0.109 | 0.742 |  |
| Duration of disease (years) | 1 | 2.8 | 2.8 | 16.603 | <0.001^**^ |  |
| Dosage of nilotinib (mg) | 1 | 0.9 | 0.9 | 5.104 | 0.025 |  |
| Barthel index score change |  |  |  |  |  |  |
| Residuals |  | 407.9 | 2.9 |  |  | – |
| Follow-up (months) | 4 | 8.4 | 2.1 | 0.730 | 0.573 |  |
| SARA score at baseline | 1 | 921.8 | 921.8 | 320.939 | <0.001^**^ |  |
| Age (years) | 1 | 1.4 | 1.4 | 0.493 | 0.484 |  |
| Male sex | 1 | 17.7 | 17.7 | 6.158 | 0.014^*^ |  |
| Type | 5 | 24.4 | 4.9 | 1.698 | 0.139 |  |
| Trinucleotide repeat length (long allele) | 1 | 2.2 | 2.2 | 0.782 | 0.378 | – |
| Duration of disease (years) | 1 | 4.5 | 4.5 | 1.564 | 0.213 | – |
| Dosage of nilotinib (mg) | 1 | 49.4 | 49.4 | 17.200 | <0.001^**^ | – |

ANCOVA: analysis of covariance, DF: degree of freedom, FARS: Rating scale for Friedreich’s ataxia, and SCA: spinocerebellar ataxia. ^**^*P*<0.01.

**Supplemental Table 3. Differentially expressed proteins among the serum of the subgroups**

| **UniprotID** | **Protein description** | **Name** | ***P*** | **Benjamini-Hochberg critical value** |
| --- | --- | --- | --- | --- |
| **Pattern 1**  **C > N > R** |  |  |  |  |
| P02750 | Leucine-rich alpha-2-glycoprotein | LRG1 | 0.0001 | 0.0002^*^ |
| P19320 | Vascular cell adhesion protein 1 | VCAM1 | 0.0043 | 0.0004 |
| P48735 | Isocitrate dehydrogenase [NADP], mitochondrial | IDH2 | 0.0073 | 0.0007 |
| Q9UM47 | Neurogenic locus notch homolog protein 3;Notch 3 extracellular truncation;Notch 3 intracellular domain | NOTCH3 | 0.0080 | 0.0009 |
| P08581 | Hepatocyte growth factor receptor | MET | 0.0172 | 0.0011 |
| Q93099 | Homogentisate 1,2-dioxygenase | HGD | 0.0208 | 0.0013 |
| P48637 | Glutathione synthetase | GSS | 0.0231 | 0.0016 |
| H0YEA7 | N-acetylglucosamine-1-phosphotransferase subunit gamma | GNPTG | 0.0246 | 0.0018 |
| P0DOY3 | Ig lambda-6 chain C region;Ig lambda-7 chain C region | IGLC6 | 0.0273 | 0.0020 |
| Q9HBR0 | Putative sodium-coupled neutral amino acid transporter 10 | SLC38A10 | 0.0279 | 0.0022 |
| P51884 | Lumican | LUM | 0.0303 | 0.0025 |
| P26639 | Threonine--tRNA ligase, cytoplasmic | TARS | 0.0318 | 0.0027 |
| Q9P232 | Contactin-3 | CNTN3 | 0.0324 | 0.0029 |
| P01834 | Ig kappa chain C region | IGKC | 0.0366 | 0.0031 |
| O15117 | FYN-binding protein | FYB | 0.0391 | 0.0034 |
| A0A7P0TAR4 | Anthrax toxin receptor 2 | ANTXR2 | 0.0402 | 0.0036 |
| P23381 | Tryptophan--tRNA ligase, cytoplasmic;T1-TrpRS;T2-TrpRS | WARS | 0.0413 | 0.0038 |
| E9PN95 | Uteroglobin | SCGB1A1 | 0.0436 | 0.0040 |
| Q14515 | SPARC-like protein 1 | SPARCL1 | 0.0451 | 0.0042 |
| P39060-2 | Collagen alpha-1(XVIII) chain;Endostatin | COL18A1 | 0.0455 | 0.0045 |
| E9PNW4 | CD59 glycoprotein | CD59 | 0.0465 | 0.0047 |
| P23142 | Fibulin-1 | FBLN1 | 0.0475 | 0.0049 |
| **Pattern 2**  **R > C > N** |  |  |  |  |
| D6RF20 | Vitamin-D binding protein | GC | 0.0002 | 0.0002^*^ |
| P17174 | Aspartate aminotransferase, cytoplasmic | GOT1 | 0.0007 | 0.0004 |
| Q9H2U2 | Inorganic pyrophosphatase 2, mitochondrial | PPA2 | 0.0081 | 0.0007 |
| A0A0U1RR32 | Histone H2A type 1-J | HIST1H2AJ | 0.0092 | 0.0009 |
| P08648 | Integrin alpha-5 | ITGA5 | 0.0148 | 0.0011 |
| P00352 | Retinal dehydrogenase 1 | ALDH1A1 | 0.0169 | 0.0013 |
| Q9UBS4 | DnaJ homolog subfamily B member 11 | DNAJB11 | 0.0177 | 0.0016 |
| Q8TDY8 | Immunoglobulin superfamily DCC subclass member 4 | IGDCC4 | 0.0228 | 0.0018 |
| P25815 | Protein S100-P | S100P | 0.0303 | 0.0020 |
| P02741 | C-reactive protein | CRP | 0.0320 | 0.0022 |
| P11142 | Heat shock cognate 71 kDa protein | HSPA8 | 0.0425 | 0.0025 |
| Q6UXK5 | Leucine-rich repeat neuronal protein 1 | LRRN1 | 0.0434 | 0.0027 |
| A0A7P0TAK8 | Neurogenic locus notch homolog protein 1 | NOTCH1 | 0.0480 | 0.0029 |
| **Pattern 3**  **R > N > C** |  |  |  |  |
| P20851-2 | C4b-binding protein beta chain | C4BPB | 0.0002 | 0.0002^*^ |
| P04003 | C4b-binding protein alpha chain | C4BPA | 0.0004 | 0.0004^*^ |
| Q02818 | Nucleobindin-1 | NUCB1 | 0.0050 | 0.0007 |
| P31327 | Carbamoyl-phosphate synthase [ammonia], mitochondrial | CPS1 | 0.0075 | 0.0009 |
| P62495-2 | Eukaryotic peptide chain release factor subunit 1 | ETF1 | 0.0088 | 0.0011 |
| A0A3B3ISJ1 | Vitamin K-dependent protein S | PROS1 | 0.0093 | 0.0013 |
| P08185 | Corticosteroid-binding globulin | SERPINA6 | 0.0113 | 0.0016 |
| Q04446 | 1,4-alpha-glucan-branching enzyme | GBE1 | 0.0116 | 0.0018 |
| P55056 | Apolipoprotein C-IV | APOC4 | 0.0137 | 0.0020 |
| P00738 | Haptoglobin;Haptoglobin alpha chain;Haptoglobin beta chain | HP | 0.0207 | 0.0022 |
| P00739 | Haptoglobin-related protein | HPR | 0.0225 | 0.0025 |
| A0A087WTA8 | Collagen alpha-2(I) chain | COL1A2 | 0.0251 | 0.0027 |
| B1AK87 | F-actin-capping protein subunit beta | CAPZB | 0.0269 | 0.0029 |
| E7END6 | Vitamin K-dependent protein C | PROC | 0.0429 | 0.0031 |
| O43157-2 | Plexin-B1 | PLXNB1 | 0.0436 | 0.0034 |

C: control, N: non-responsive to nilotinib, and R: responsive to nilotinib. Benjamini-Hochberg adjustment was used for the adjustment for the multiple comparison. Benjamini-Hochberg critical value was calculated using the false discovery rate of 0.2. ^*^Statistical significance after Benjamini-Hochberg adjustment.

**Supplemental Table 4. Subjects with serum proteomic analysis**

| Number | Response | Median Age of each group (years) | Type / Symptom | Trinucleotide repeat length  (long allele) | Median Age of onset of each group (years) | Dosage of nilotinib (mg/day) | SARA score at baseline | SARA score at 12M | SARA score change-12M |
| --- | --- | --- | --- | --- | --- | --- | --- | --- | --- |
| R01 | Yes |  | SCA7 | 45 |  | 300 | 26 | 22 | 4 |
| R02 | Yes |  | SCA2 | 46 |  | 300 | 14 | 7 | 7 |
| R03 | Yes | 36 | SCA3 | 73 | 31 | 300 | 10 | 6 | 4 |
| R04 | Yes |  | SCA2 | 45 |  | 150 | 15 | 11 | 4 |
| R05 | Yes |  | SCA8 | 81 |  | 300 | 10.5 | 7.5 | 3 |
| N01 | No |  | SCA3 | 71 |  | 300 | 17 | 19 | -2 |
| N02 | No |  | SCA6 | 24 |  | 300 | 5.5 | 9 | -3.5 |
| N03 | No | 50 | SCA3 | 76 | 43 | 300 | 10.5 | 12 | -1.5 |
| N04 | No |  | SCA8 | 108 |  | 300 | 9 | 11 | -2 |
| N05 | No |  | SCA6 | 22 |  | 200 | 5.5 | 13 | -7.5 |
| C01 | – |  | Dizziness | – | – | – | – | – | – |
| C02 | – |  | Dizziness | – | – | – | – | – | – |
| C03 | – | 44 | Dizziness | – | – | – | – | – | – |
| C04 | – |  | Dizziness | – | – | – | – | – | – |
| C05 | – |  | Dizziness | – | – | – | – | – | – |

SCA: spinocerebellar ataxia and SARA: scale for the assessment and rating of ataxia
